# Supplementary material for: Diagnosing Systemic Disorders with AI Algorithms Based on Ocular Images
Source: Healthcare (Basel). 2023 Jun 13;11(12):1739. doi: 10.3390/healthcare11121739 (PMC10298137; doi:10.3390/healthcare11121739)
Supplement: Supplementary file 1 [file healthcare-11-01739-s001.zip › PRISMA.pdf]

Supplementary Table S1. The search strategy used for obtaining research articles in the three selected databases

Database	step	Component	Search Terms	Result
Pubmed	1	Artificial intelligence	(artificial intelligence OR machine learning OR deep learning OR neural network)	313,071
	2	Ocular images	((eye) OR (ocular) OR (fundus) OR (retina) OR (ophthalmology) OR (optical coherence tomography) OR (slip-lamp microscopy)) AND ((image) OR (photography) OR (picture))	141,279
	3	Systemic Diseases	(((cardiovascular diseases [MeSH Terms]) OR (Kidney Diseases [MeSH Terms]) OR (Neurodegenerative Diseases [MeSH Terms]) OR (System Diseases [MeSH Terms]) OR (Psychiatric Diseases [MeSH Terms]) OR (Hepatobiliary Diseases [MeSH Terms]) OR (Hematologic Diseases [MeSH Terms])) AND (eye [MeSH Terms]))	36,260
	4	1 AND 2 AND 3 Filters	Full text, Humans, English, Exclude preprints, publication date 2013/1/1 - 2023/3/1	323
Web of Science	1	Artificial intelligence	((((TS=(artificial intelligence)) OR TS=(deep learning))) OR TS=(machine learning)) OR TS=(neural network)	1,759,777
	2	Ocular images	(((((((TS=(eye)) OR TS=(ocular)) OR TS=(ophthalmic)) OR TS=(retina)) OR TS=(fundus)) OR TS=(optical coherence tomography)) OR TS=(slip-lamp microscopy)) AND (TS=(image) OR TS=(picture) OR TS=(photography))	374,508

	3	Systemic Diseases	((TS=(systemic diseases) OR TS=(cardiovascular diseases) OR TS=(neurodegenerative diseases) OR TS=(psychiatric diseases) OR TS=(kidney disease) OR TS=(hematological disease) OR TS=(hepatobiliary diseases) OR TS=(respiration system disease) OR TS=(digestive disease))AND TS=(eye))	173,590
	4	Others	Refined By: Languages: English. NOT Document Types: Meeting or Review Article or Editorial Material or Book or Unspecified or Patent or Letter or Preprint or Case Report	506
	5	4 Filters	[publication date 2013/1/1 - 2023/3/1]	446
Embase	1	Artificial intelligence	('artificial intelligence'/exp/mj OR 'machine learning'/exp/mj OR 'deep learning'/exp/mj OR 'neural network'/exp/mj)	169,909
	2	Ocular images	(‘eye'/exp/mj OR 'ophthalmology'/exp OR ‘ocular’/exp OR 'fundus '/exp OR 'retina'/exp OR 'optical coherence tomography'/exp OR 'slit lamp microscopy'/exp) AND (‘image’/exp OR ‘picture’/exp OR ‘photography’/exp)	15,479
	3	Systemic Diseases	(‘systemic diseases’/exp/mj OR ‘cardiovascular diseases’/exp/mj OR ‘neurodegenerative diseases’/exp/mj OR ‘psychiatric diseases’/exp/mj OR ‘ kidney disease’/exp/mj OR ‘hematological disease’/exp/mj OR ‘hepatobiliary diseases’/exp/mj OR ‘respiration system disease’/exp/mj OR ‘digestive disease’/exp/mj) AND ‘eye’/exp/mj)	19,231
	4	1 AND 2 AND 3 Filters	[01-01-2013]/sd NOT [02-03-2023]/sd	28
