# Supplementary material for: Diagnosing Systemic Disorders with AI Algorithms Based on Ocular Images
Source: Healthcare (Basel). 2023 Jun 13;11(12):1739. doi: 10.3390/healthcare11121739 (PMC10298137; doi:10.3390/healthcare11121739)
Supplement: Supplementary file 1 [file healthcare-11-01739-s001.zip › QUADAS-2.pdf]

Supplementary Table S2. The detailed result of the QUADAS-2 analysis.

Author, Year	RISK OF BIAS				APPLICABILITY CONCERNS		
	PATIENT SELECTION	INDEX TEST	REFERENCE STANDARD	FLOW AND TIMING	PATIENT SELECTION	INDEX TEST	REFERENCE STANDARD
Cheung et al. 2021	☺	☺	☺	☺	☺	☺	☺
Lee et al. 2023	☺	☺	☺	☺	☺	☺	☺
Son et al. 2020	☺	☺	☺	☺	☺	☹	☺
Poplin et al. 2018	☺	☺	☺	☺	☺	☺	☺
Nusinovici et al. 2022	☺	☺	☺	☺	☺	☺	☺
Rim et al. 2021	☺	☺	☺	☺	☺	☺	☺
Tseng et al. 2023	☺	☺	☺	☺	☺	☺	☺
Chang et al. 2020	☺	☺	☺	☺	☺	☺	☺
Duan et al. 2022	☹	?	☹	☺	☹	?	☹
Diaz-Pinto et al. 2022	☺	☺	☺	☺	☺	☺	☺
Zekavat et al. 2022	☺	☺	☺	☺	☺	☺	☺
Nunes et al. 2019	☹	☹	☹	☺	☹	☹	☹
Wang et al. 2022	☹	☺	☺	☺	☹	☺	☺
Wang et al. 2022	☹	☺	☺	☺	☹	☺	☺
Xie et al. 2023	☹	☺	☺	☺	☹	☺	☺

Tian et al. 2021	☺	☺	☺	☺	☺	☺	☺
Cheung et al. 2022	☺	☺	☺	☺	☺	☺	☺
Ahn et al. 2023	☹	☺	☺	☺	☹	☺	☺
Hu et al. 2022	☺	☺	☺	☺	☺	☺	☺
Montolío et al. 2021	☹	☺	☺	☺	☹	☺	☺
Pérez Del Palomar et al. 2019	☹	☺	☺	☺	☹	☺	☺
Appaji et al. 2022	☹	☹	☹	☺	☹	☹	☹
Sabanayagam et al. 2022	☺	☺	☺	☺	☺	☺	☺
Zhang et al. 2021	☺	☺	☺	☺	☺	☺	☺
Zhang et al. 2022	☺	☺	☺	☺	☺	☺	☺
Bauskar et al. 2019	☹	☺	☺	☺	☹	☺	☺
Chen et al. 2022	☺	☺	☺	☺	☺	☺	☺
Mitani et al. 2020	☺	☺	☺	☺	☺	☺	☺
Zhao et al. 2022	☺	☺	☺	☺	☺	☺	☺
Wei et al. 2021	☺	☺	☺	☺	☺	☺	☺
Wu et al. 2022	☹	☺	☺	☺	☹	☺	☺
Xiao et al. 2021	☺	☺	☺	☺	☺	☺	☺
Huang et al. 2023	☺	☺	☺	☺	☺	☺	☺
Lv et al. 2021	☹	☺	☺	☺	☹	☺	☺
Rim et al. 2020	☺	☺	☺	☺	☺	☺	☺

Kim et al. 2022	☺	☺	☺	☺	☺	☺	☺

☺Low Risk ☹High Risk ? Unclear Risk
