# Supplementary figures and images for: Diagnosing Systemic Disorders with AI Algorithms Based on Ocular Images
Source: Healthcare (Basel). 2023 Jun 13;11(12):1739. doi: 10.3390/healthcare11121739 (PMC10298137; doi:10.3390/healthcare11121739)

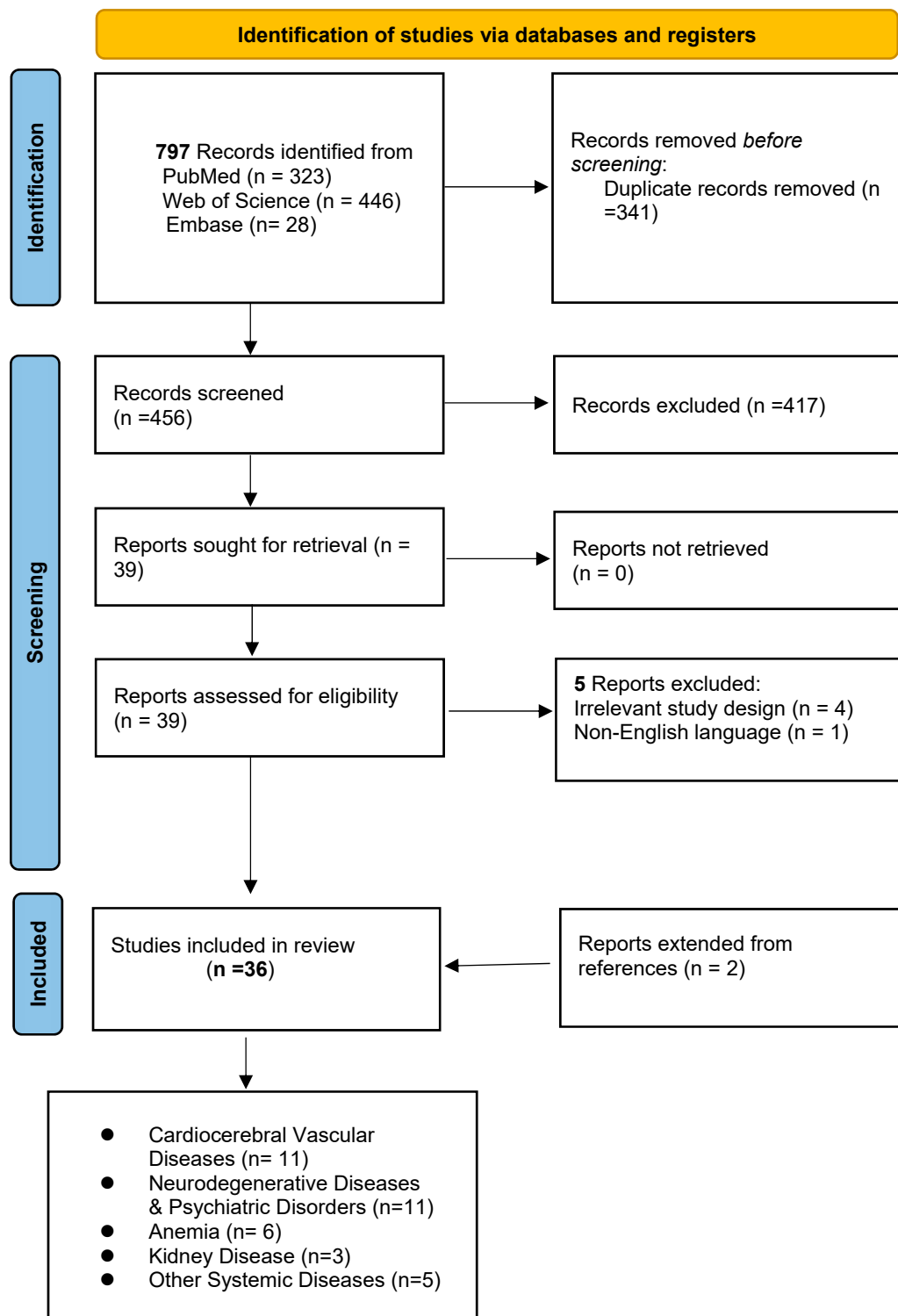

**Supplementary Figure S1.** Flowchart of the study screening process.

Supplement: Supplementary file 1 [file healthcare-11-01739-s001.zip › flowchart.pdf]
